# Mental health literacy survey among Cambodia’s urban and rural populations: Results from a vignette-based population survey

**DOI:** 10.1371/journal.pone.0265120

**Published:** 2022-04-28

**Authors:** Akihiro Nishio, Toshiyuki Marutani

**Affiliations:** 1 Health Administration Center, Gifu University, Gifu, Japan; 2 Department of Psychopathology, Division of Neuroscience, Graduate School of Medicine, Gifu University, Gifu, Japan; 3 Health Support Center, Tokyo Institute of Technology, Yokohama, Japan; St John’s University, UNITED KINGDOM

## Abstract

**Background:**

Although there are effective methods for the treatment and management of various mental illnesses, some individuals still do not seek psychiatric treatment. Various factors could affect this reluctance toward treatment, one of which is the public’s lack of mental health literacy. This survey aimed to measure and compare mental health literacy in Cambodia’s urban and rural areas.

**Method:**

Tours were held to hold seminars that provided information about mental health in the health centers around Phnom Penh (PP) and Siem Reap (SR), and a survey was conducted on mental health literacy for the participants before the seminar at each location. Anthony Jorm’s vignette of psychosis (young adult) and Angermeyer’s questionnaire were used. After the participants were classified into the “agree group” and “disagree group” for each item, the answers for each item given by the participants in PP and SR were compared using the chi-square test and the odds rate was calculated.

**Results:**

The participants in SR were more likely to give reasons such as inherited causes, economic problems, stress at work, or family problems as the cause of schizophrenia. The percentage of these beliefs about schizophrenia was relatively lower in PP than in SR. Regarding attitudes toward schizophrenia, the participants in SR were more likely to have negative views and predict negative prognoses than the participants in PP. As for participants’ feelings about schizophrenia, the participants in SR reacted more strongly than those in PP. Even though the participants in SR reacted more negatively, they were sympathetic toward individuals with schizophrenia.

**Conclusion:**

Overall, the participants in the SR group were more likely to have negative attitudes toward schizophrenia than those in the PP group. These results support our hypothesis that mental health literacy represents the maturity of community mental health in a targeted area.

## Background

Although there are effective methods for the treatment and management of various mental illnesses, some individuals still do not seek psychiatric or psychological treatment. Several factors may affect this reluctance toward treatment, one of which is the public’s lack of mental health literacy, specifically a lack of knowledge on how to recognize mental illness and beliefs about treatment that differ from those of health professionals [1, 2]. In places where people have low mental health literacy, they may face difficulties in seeking professional help and may tend to seek help much later than those who have high mental health literacy. Delays in receiving treatment are associated with poorer outcomes for mental illness [3, 4]. Since poor outcomes may lead to a negative image of mental health treatment, the stigma toward mental illness or low mental health literacy is reinforced by this cycle. Mental health literacy depends on the individual’s perception. However, by obtaining an adequate sample, the average mental health literacy could represent the maturity of community mental health in the targeted areas. Mental health literacy is vital for evaluating the maturity of community mental health.

Although there are several scales that measure mental health literacy, a vignette-style survey was chosen for this study. The reasons were: (1) residents of developing countries are not accustomed to answering abstract questionnaires, (2) some specific terms cannot be translated into local languages, and (3) a vignette-style survey can reflect general attitudes related to diverse aspects of mental illness or treatment. Anthony Jorm’s [5] concept of mental health literacy was used as the theoretical framework to evaluate the public’s knowledge, attitudes, and beliefs about mental illness. Mental health literacy refers to “knowledge and beliefs about mental disorders which aid their recognition, management, or prevention” and includes “the ability to recognize specific disorders; knowing how to seek mental health information; knowledge of risk factors and causes, of self-treatments, and of professional help available” [6]. The presence of this knowledge may play an important role in seeking help for mental health problems [7].

The aim of this study was to determine the state of mental health literacy in the urban zones in Phnom Penh (PP) and the rural parts of Siem Reap (SR) in Cambodia. This comparison demonstrates our hypothesis that the urbanization of Cambodian people and the large gap in the provision of mental health services has impacted their mental health literacy. This study might promote the enhancement of programs for mental health literacy for Cambodian citizens in the future.

## Methods

### Participants

The survey was conducted in PP and SR in Cambodia from 2016 to 2017. Tours were taken to hold seminars that provided information about mental health in the health centers around PP and SR, and a survey was conducted on mental health literacy for the participants before the seminar at each location. The seminar took about one hour. The characteristics of schizophrenia, differences from other psychotic diseases, and treatment for schizophrenia were mentioned. The total number of participants was 131 in PP and 215 in SR. The inclusion criteria of participants were Cambodian who participated in our seminar. Those who were under 18 years old, and those who did not answer questions about their age or gender were excluded. However, participants who did not answer several questions were not excluded. Thus, the total number of participants was different for each item.

Cambodia is located in the southern part of the Indochina Peninsula in Southeast Asia and has a population of over 16 million. Cambodia has struggled with many political and economic difficulties; however, after more than two decades of strong economic growth, Cambodia became a lower middle-income country in 2015. The gross national income per capita (current international USD) was 1,530 USD in 2019 [8].

PP, which has around two million inhabitants, is the capital of Cambodia. At the time of the study, there were four facilities that provided psychiatric consultations in PP: Khmer Soviet Friendship Hospital, Kossamak Hospital, Phnom Penh Municipal Hospital, and TPO clinics. Only the Khmer Soviet Friendship Hospital had inpatient wards. However, this scale was dependent on the budget and was unstable. The fee for mental health services for outpatients was not covered by national health insurance, and it ranged from 5 to 40 USD in PP.

With a population of about 1.4 million in 2019, SR is the fifth largest province in Cambodia [9]. At the time of our study, SR had one central hospital in the city of Siem Reap and three district hospitals in the Angkor Chum, Kralanh, and Sout Nikom districts. Only the central hospital had two staff psychiatrists. The other three hospitals provided psychiatric services twice a month, with doctors traveling from the central hospital in SR or other provincial hospitals. No inpatient hospitalization settings were available for patients with psychosis in SR. Treatment was free for low-income patients who were recognized through the “Identification of Poor Households” program [10]. If patients were extremely poor, the traffic fee (1 USD) was paid for them. Patients with a middle or high economic status had to pay 5 USD for each visit, which included the cost of medication. Only one-third of the patients were economically stable. Further, this program was shut down after our survey due to a lack of funds. Following some changes to the system, only extremely patients poor are presently exempt from paying for treatment.

### The vignettes and the questionnaire

In this study, Anthony Jorm’s vignette of psychosis (young adults) [11], published in 2007, was used. The symptoms described in the vignettes fulfill the criteria of the Diagnostic and Statistical Manual of Mental Disorders (DSM, 5^th^ edition) for schizophrenia [12]. The original version of Anthony Jorm’s survey was an open-ended questionnaire. Since it was considered difficult to analyze the answers of participants in the Cambodian language, Khmer, Angermeyer’s questionnaire [13] was chosen. In the questionnaire, following the presentation of the vignette, the respondents were asked whether, according to them, the problem depicted in each respective vignette denoted “a mental illness in the medical sense.” Next, the respondents’ causal attributions were elicited using a list of 12 possible causes. Further, the respondents’ attitudes toward this case and treatment were elicited. Help-seeking recommendations were assessed using a catalog of the various sources of help available. Finally, participants’ feelings about this case were measured using eight questions. The vignettes, questions, and answers were translated into Khmer from English by a certified professional Cambodian translator. The translated questionnaire was distributed to each participant. All sentences were read out loud, and the research assistants helped those who had trouble understanding them. After the presentation of the vignettes depicting a person with schizophrenia, the participants responded to the questionnaire in which the items were scored on a 5-point Likert scale: “strongly agree” and “agree” were classified as “agree,” whereas “neutral,” “disagree,” and “strongly disagree” were classified as “not agree.”

### Analysis

Since most of the participants could not fully answer the questionnaire despite support from the research assistants, participants who did not complete the questionnaire were not excluded. The number of respondents was different for each item. However, respondents who did not provide information about their age or gender were excluded. After classifying the participants into the “agree group” and “not agree group” for each item in PP and SR, the answers for each item given by the participants in PP and SR were compared using the chi-square test, and the odds rate for each item was calculated. Statistical analyses were performed using JMP® version 10.0.2 (SAS Institute, Tokyo, Japan).

### Ethical considerations

The research design was approved by the Ethical Review Committee of the Graduate School of Medicine, Gifu University, in 2015 (approval No. 27–134) and the Ethical Committee of the Ministry of Health in Cambodia in 2016 (approval No. 031, NECHR). All participants received detailed face-to-face explanations regarding the protocol before providing their consent. The consent form was written in an easy-to-read manner. All participants gave written informed consent.

## Results

The number of men and women in PP was 56 (45.5%) and 75 (54.5%), respectively. The number of men and women in SR was 104 (48.4%) and 111 (51.6%), respectively. The average ages of the participants in PP and SR were 27.9 and 44.2 years, respectively. The survey was conducted in the same setting for PP and SR. However, the average ages of the participants belonging to PP and SR were significantly different.

Table 1 outlines the participants’ basic understanding of schizophrenia in PP and SR. Participants from both areas had a similar understanding and any significant differences were not found in their responses.

**Table 1:** Participants’ basic understanding of schizophrenia.

	Phnom Penh (N = 131)		Siem Reap (N = 215)		Odds	
	Agree (%)	Not Agree (%)	Agree (%)	Not Agree (%)	Odds ratio	P
This is a physical illness.	25 (19.8)	101 (80.2)	34 (16.5)	172(83.5)	0.799	0.462
This is a mental illness.	114 (88.4)	15 (11.6)	171 (82.2)	37 (17.8%)	0.608	0.162
This a reaction to stress.	40 (31.3)	88 (68.8)	80 (37.2)	135 (62.8)	1.304	0.293

The chi-square test was conducted and the odds rate was calculated for each item.

*p<0.05

**p<0.01.

Table 2 presents the participants’ causal attributions for schizophrenia in PP and SR. The participants in SR were more likely to agree with statements such as “He inherited this problem from his family” (OR = 2.653, p = 0.002) or “His problem is caused by bad blood in his family” (OR = 3.122, p = 0.000) than those in PP. Further, the participants in SR were more likely to agree with causal attributions related to the environment such as, “This problem is caused by worries about the family/partnership” (OR = 1.786, p = 0.011), “He experiences stress at work” (OR = 2.045, p = 0.002), “He has a problem because he was raised by bad parents” (OR = 1.858, p = 0.013), “His problem is caused by being poor” (OR = 2.957, p = 0.000), and “His problem is due to sexual abuse in childhood” (OR = 2.618, p = 0.000) than those in PP. In sum, the participants in SR were more likely to understand that schizophrenia could be caused due to environmental or inherited attributes compared to those in PP.

**Table 2:** Participants’ causal attributions for schizophrenia.

	Phnom Penh (N = 131)		Siem Reap (N = 215)		Odds	
	Agree (%)	Not Agree (%)	Agree (%)	Not Agree (%)	Odds ratio	P
This problem is caused by a brain disease.	72 (55.4)	58 (44.6)	134 (62.6)	80 (37.4)	1.349	0.212
He inherited this problem from his family.	14 (10.9)	115 (89.1)	52 (24.4)	161 (75.6)	2.653	0.002**
This problem is caused by worries about the family/partnership.	54 (41.5)	76 (58.5)	118 (55.9)	93 (44.1)	1.786	0.011*
This problem is caused by a negative life event.	85 (67.5)	41 (32.5)	126 (59.4)	86 (40.6)	0.707	0.164
This person experiences stress at work.	45 (35.2)	83 (64.8)	112 (52.6)	101 (47.4)	2.045	0.002*
He has a problem because he was raised by bad parents.	34 (27.6)	89 (72.4)	88 (41.5)	124 (58.5)	1.858	0.013*
This person’s problem is due to a lack of parental affection.	53(41.7)	74 (58.3)	96 (45.1)	117 (54.9)	1.146	0.573
This person’s problem is caused by bad blood in his family.	12 (9.4)	116 (90.6)	52 (24.4)	161 (75.6)	3.122	0.000**
This person’s problem is caused by being poor.	33 (26.0)	94 (74.0)	109 (50.9)	105 (49.1)	2.957	0.000**
This person must have done something wrong.	53 (41.4)	75 (58.6)	105 (49.3)	108 (50.7)	1.376	0.179
His problem is due to sexual abuse in childhood.	28 (21.7)	101 (78.3)	90 (42.1)	124 (57.9)	2.618	0.000**
This person’s problem is caused by a lack of willpower	67 (51.9)	62 (48.1)	124 (58.5)	88 (41.5)	1.304	0.261

The chi-square test was conducted and the odds rate was calculated for each item.

*p<0.05

**p<0.01.

Table 3 outlines the participants’ attitudes toward patients with schizophrenia and its treatment. Except for the final question in this series, the participants expressed negative views of schizophrenia for all items. The participants in SR were more likely to agree with items showing negative images or a negative prognosis of schizophrenia, such as “Even with treatment, such individuals will not change significantly” (OR = 2.352, p = 0.000), “Such individuals bring shame to their families” (OR = 3.828, p = 0.000), “Such individuals are a bad influence on their society” (OR = 2.916, p = 0.000), “Such individuals are different from others” (OR = 1.987, p = 0.005), “Once you encounter such problems, you cannot return to a normal productive life” (OR = 2.448, p = 0.000), and “Such individuals are a danger to the society” (OR = 3.744, p = 0.000) than those in PP. Overall, negative attitudes toward schizophrenia were predominant in the SR region. However, for the item, “Such individuals should not have kids because they will pass on their bad genes,” participants showed lenient attitudes toward schizophrenia in both SR and PP.

**Table 3:** Participants’ attitudes toward schizophrenia and treatment.

	Phnom Penh (N = 131)		Siem Reap (N = 215)		Odds	
	Agree (%)	Not Agree (%)	Agree (%)	Not Agree (%)	Odds ratio	p
Even with treatment, such individuals will not change significantly.	39 (30.2)	90 (69.8)	106 (50.5)	104 (49.5)	2.352	0.000**
Such individuals are responsible for their condition.	70 (55.1)	57 (44.9)	91 (43.3)	119 (56.7)	0.623	0.043
Such individuals should not have kids because they will pass on their bad genes.	24 (19.0)	102 (81.0)	44 (21.0)	166 (79.0)	1.127	0.779
Such individuals bring shame to their families.	24 (18.6)	105 (81.4)	98 (46.7)	112 (53.3)	3.828	0.000**
Such individuals are a bad influence on their societies.	32 (25.0)	96 (75.0)	104 (49.3)	107 (50.7)	2.916	0.000**
Such individuals are different from others.	77 (60.2)	51 (39.8)	159 (75.6)	53 (25.0)	1.987	0.005**
Once you encounter such problems, you cannot return to a normal productive life.	30 (23.3)	99 (76.7)	89 (42.6)	120 (57.4)	2.448	0.000**
Such individuals tend to commit crimes.	86 (66.7)	43 (33.3)	134 (62.9)	79 (37.1)	0.848	0.561
Such individuals are a danger to their societies.	36 (28.1)	92 (71.9)	126 (59.4)	86 (40.6)	3.744	0.000**
Basically, we are all sometimes like this person. It’s just a question of how pronounced this state is.	67 (52.3)	61 (47.7)	136 (63.8)	77 (36.2)	1.62	0.040*

The chi-square test was conducted and the odds rate was calculated for each item.

*p<0.05

**p<0.01.

Table 4 displays the participants’ help-seeking recommendations for schizophrenia. The participants in PP were more likely to recommend consulting a psychiatrist or psychologist versus other medical professionals or religious leaders. In SR, participants suggested seeking help not only from psychiatrists, but also from other medical professionals or religious leaders. Psychological treatment was not available in the SR region at the time of our survey. However, 66.5% of the participants in SR agreed to seek help from a psychologist. This could be because after the civil war, many international organizations offered psychological treatment in SR and people remembered that, or perhaps the term “psychologist” was misinterpreted by participants in the SR group. Furthermore, the participants in PP were more likely to agree with statements such as “He can deal with it by himself” (OR = 0.396, p = 0.001) than those in SR.

**Table 4:** Participants’ help-seeking recommendations.

	Phnom Penh (N = 131)		Siem Reap (N = 215)		Odds	
	Agree (%)	Not Agree (%)	Agree (%)	Not Agree (%)	Odds ratio	p
Psychiatrist	79 (65.3)	42 (34.7)	184 (87.2)	27 (12.8)	3.623	0.000**
Psychologist	84 (72.7)	30 (26.3)	135 (66.5)	68 (33.5)	0.709	0.206
General practitioner	30 (26.5)	83 (73.5)	130 (64.7)	71 (35.3)	5.066	0.000**
Pastor/priest	45 (39.8)	68 (60.2)	116 (59.5)	79 (40.5)	2.219	0.001**
Health worker (Nurse)	22 (19.5)	91 (80.5)	133 (67.2)	65 (32.8)	8.464	0.000**
Close friends	59 (50.9)	57 (49.1)	115 (58.1)	83 (41.9)	1.339	0.24
He can deal with it by himself	37 (32.2)	78 (67.8)	31 (15.8)	165 (84.2)	0.396	0.001**

The chi-square test was conducted and the odds rate was calculated for each item.

*p<0.05

**p<0.01.

Finally, participants’ feelings about this case are depicted in Table 5. Significant differences were noted in that the participants in SR reacted more strongly than those in PP. Participants in SR reacted negatively to schizophrenia by agreeing to statements such as “I feel uncomfortable” (OR = 2.428, p = 0.000), “He makes me feel insecure” (OR = 3.362, p = 0.000), “I feel annoyed by him” (OR = 5.318, p = 0.000), and “I react angrily” (OR = 2.263, p = 0.016) more than the participants in PP. However, the participants in SR reacted to schizophrenia in a more sympathetic way by agreeing to statements such as “I feel pity for him” (OR = 2.648, P = 0.007) and “I feel sympathy for him” (OR = 4.679, p = 0.000) when compared to the participants in PP.

**Table 5:** Participants’ feelings about schizophrenia.

	Phnom Penh (N = 131)		Siem Reap (N = 215)		Odds	
	Agree (%)	Not Agree (%)	Agree (%)	Not Agree (%)	Odds ratio	p
I feel the need to help him.	116 (89.2)	14 (10.8)	192 (91.4)	18 (8.6)	1.287	0.568
I feel pity for him.	108 (83.1)	22 (16.9)	195 (92.9)	15 (7.1)	2.648	0.007**
I feel sympathy for him.	73 (56.6)	56 (43.4)	183 (85.9)	30 (14.1)	4.679	0.000**
I feel uncomfortable.	49 (38.3)	79 (61.7)	128 (60.1)	85 (39.5)	2.428	0.000**
He makes me feel insecure.	44 (34.1)	85 (65.9)	134 (63.5)	77 (36.5)	3.362	0.000**
I feel annoyed by him.	11 (8.6)	117 (91.4)	71 (33.3)	142 (66.7)	5.318	0.000**
I react angrily.	13 (10.3)	113 (89.7)	44 (20.7)	169 (79.3)	2.263	0.016*
I am amused by something like that.	14 (10.9)	114 (89.1)	45 (21.1)	168 (78.9)	2.181	0.018*

The chi-square test was conducted and the odds rate was calculated for each item.

*p<0.05

**p<0.01.

## Discussion

This is the first survey to investigate mental health literacy in Cambodia and compare the differences between urban zones (PP) and rural areas (SR). We found that Cambodian people viewed schizophrenia differently depending on where they resided. Participants in rural regions were more likely to attribute reasons such as inherited causes, economic problems, stress at work, or family problems as the root of schizophrenia. The percentage of these beliefs about schizophrenia was relatively lower in PP than in SR. Regarding attitudes toward schizophrenia, the participants in SR were more likely to hold negative views and to predict negative prognoses than the participants in PP. As for help-seeking recommendations for schizophrenia, the participants in PP were more likely to recommend consulting a psychiatrist or psychologist versus other medical professionals or religious leaders. In SR, the participants suggested seeking help not only from a psychiatrist, but also from other medical professionals or religious leaders. In terms of participants’ feelings about schizophrenia, the participants in SR reacted more strongly than those in PP. Even though the participants in SR reacted more negatively, they were also sympathetic toward individuals with schizophrenia. The large gap in the availability of psychiatric services between these two areas may have led to differences in knowledge about mental illness.

A major concern in this study was the large gap in the average age of the participants belonging to PP and SR. We conducted the survey in the same manner in PP and SR by holding lectures about mental illness at health centers around the hospital and distributing the questionnaire before the lecture. A large gap in the participants’ average age probably occurred because farming is a predominant occupation in SR [14], and middle-aged farmers could easily attend the lecture after completing their morning work on the farm. In contrast, most middle-aged individuals in PP might be working in companies or shops during the day, and individuals belonging to the younger generation, such as students or unemployed individuals, could easily attend the seminar. To evaluate the impact of this gap in average age, the correlation rate of age with the sum of scores of all items in Table 3 was compared for PP and SR, except for the final question. These questions were chosen because the items in Table 3 consist only of negative statements about schizophrenia, except for the final question. Hence, the relationship between age and attitudes toward schizophrenia was explored using this score. The following method was employed to obtain the sum of scores: “strongly disagree” was scored as “1,” “disagree” as “2,” “neutral” as “3,” “agree” as “4,” and “strongly agree” as “5.” The average summed up scores of Table 3 (except for the last question) were 24.6 for the PP group and 28.0 for the SR group. The correlation rates between age and scores were 0.262 for PP and 0.226 for SR. This means that age has a slightly negative impact on attitudes about schizophrenia in PP and SR. However, this impact may be very limited.

Only one Chinese study has compared mental health literacy between urban and rural areas in developing countries [15], and showed that participants living in urban zones may presume a diagnosis of mental illness more accurately than those who live in rural regions. In addition, many studies have harnessed diverse tools to gauge measure mental health literacy in developing countries. However, most studies have conducted interviews or used questionnaires to examine medical professionals, teachers, students, patients, and family members of patients with mental illness. Surveys targeting the general population are limited. Some surveys have reported lower recognition of mental illness among Chinese people compared to people in Western countries, depending on participants’ sociodemographic status [16–20]. In these surveys, the participants were shown several vignettes, after which they had to choose the diagnosis they believed to be applicable. Besides China, reports from India [21], Turkey [22], South Africa [23], and Bangladesh [24] also exist. In the Indian survey, an open and close-ended question method (the original version of Jorm’s study) was used. In the Turkish survey, stigma among participants related to schizophrenia and depression was evaluated. The South African survey revealed that mental illness was related to stress or the patient’s will among general population. The survey in Bangladesh was tied to the relationship between the recognition of mental illness and participants’ socio-demographic status. Overall, each study had a method or research purpose that differed from those of the current study. Thus, the results of this study could not be compared with those from other countries.

Since the questionnaire created by Angermeyer for French citizens [13] was used in this study, its results could be compared with those of a survey conducted in France. Upon comparing causal beliefs about schizophrenia between Cambodian and French people, French individuals were found to attribute mental illness to factors such as family trouble (43.5%), work stress (47.0%), broken homes (21.9%), a lack of parental affection (24.4%), sexual abuse in childhood (24.4%), and weak willpower (34.5%). These rates were significantly lower for French (versus Cambodian) individuals in both PP and SR. All the responses given by French participants concerning the expected prognosis of schizophrenia were significantly more positive than those given by participants in Cambodia in both PP and SR. For instance, scores on items concerning the expected prognosis—such as “Treatment will not change the condition significantly” (7.0%), “This person is dangerous” (36.1%), and “This person is different” (38.2%)—varied significantly. In terms of help-seeking recommendations for schizophrenia, no significant differences were found between the responses of participants in France and those in Cambodia. The only difference was that French individuals were less likely to ask for help from religious leaders (9.5%). The responses of French individuals about emotional reactions were as follows: “I feel the need the help/him/her” (72.1%), “I feel pity for him” (70.9%), “I feel sympathy for him” (46.4%), “I feel uncomfortable” (57.9%), “He makes me feel insecure” (48.0%), “I feel annoyed by him” (6.5%), and “I react angrily” (5.1%). For every item, the emotional reaction to the person with schizophrenia among French individuals was weaker than that of Cambodian individuals in both PP and SR.

Overall, the participants in the SR group were more likely to have negative views of schizophrenia than those in the PP group. A similar pattern was found between the PP group and France. In other words, participants in France were less likely to have negative attitudes about schizophrenia than those in the PP group. These results support our hypothesis that mental health literacy represents the maturity of community mental health in a targeted area. From this study, it can be concluded that there is a developmental stage of mental health literacy. Therefore, mental health literacy could be used to evaluate the maturity of the mental health system. For future studies, in order to use mental health literacy as an index of the mental health system, data should be collected using a quantitative scale of mental health literacy. Further, future research should focus not only on knowledge of mental health diseases, but also on feelings or attitudes toward mental illness to choose an appropriate mental health literacy scale. This study highlights gaps in attitudes towards mental illness in developing countries.

### Limitations

There were several limitations in this study. First, there was a huge gap of average age between the participants in PP and SR. The correlations between aging and the questionnaire scores were analyzed, and it was confirmed that this age gap had a slight impact on the participants’ responses. However, the data should be considered after adjusting for the participants’ ages. Second, data were collected from a sample in a rural area, SR. While SR is a rural place, it is unlike the other rural parts of Cambodia as it contains the famous Angkor Wat ruins; therefore, SR attracted lots of international support and people had access to mental health support after the civil war. However, SR is the sixth poorest province in Cambodia [25]. Therefore, SR might represent the situation of a typical rural area in Cambodia. Third, the attitudes of individuals from urban and rural backgrounds toward cases of schizophrenia were compared. Attitudes toward cases of depression might reveal different outcomes. Thus, future studies should compare attitudes about depression with the attitudes about schizophrenia measured in this survey.

This study was the first to report differences in attitudes toward mental illness in urban and rural areas in a developing country. It also demonstrated the importance of using mental health literacy data to evaluate the maturity of mental health in targeted areas. This study’s findings will help to establish new fields in the context of global mental health research.
